# Supplementary material for: A deubiquitylase with an unusually high-affinity ubiquitin-binding domain from the scrub typhus pathogen Orientia tsutsugamushi
Source: Nat Commun. 2020 May 11;11:2343. doi: 10.1038/s41467-020-15985-4 (PMC7214410; doi:10.1038/s41467-020-15985-4)
Supplement: Supplementary file 1 — Supplementary Information [file 41467_2020_15985_MOESM1_ESM.pdf]

A Deubiquitylase with an Unusually High-affinity Ubiquitin-binding Domain from the Scrub Typhus Pathogen *Orientia tsutsugamushi*

Berk and Lim et al.

Supplementary Note 1

Initially, to determine the ubiquitin-linkage type targeted by OtDUB, we tested $OtDUB_{1-311}$ (0.5 µM) against a full panel of di-ubiquitin linkages (1 µM) and by immunoblotting did not detect any mono-ubiquitin product (Supplementary Figure 1a). The absence of mono-ubiquitin in some of our blotting assays together with the efficient cleavage of Ub-AMC (detected by a fluorescent signal) was confusing and led us to reassess di-ubiquitin cleavage using a SYPRO Ruby protein stain for detection. With this reagent, mono-ubiquitin product was detected on our SDS gels, as shown in multiple figures here. It is likely that inefficient di-ubiquitin cleavage by $OtDUB_{1-311}$ (Supplementary Figure 1a), combined with some of the mono-ubiquitin product passing through the PVDF membrane during transfer, prevented detection by anti-ubiquitin immunoblotting.

Cleavage assays using immunoblotting did help us initially identify a likely ubiquitin-binding domain (UBD) within OtDUB. For this we had used a high concentration of enzyme (0.5 µM) compared to substrate (1.0 µM) to ensure we observed activity, which actually resulted in auto-inhibition and incomplete substrate cleavage, especially for K63 chains. We anticipated that the reaction products could be inhibiting OtDUB; however, when residues 1-311 were pre-incubated with mono- (shown) or di-ubiquitin (not shown), the DUB became more active, not less, and could cleave chains to near completion within 10 min (Supplementary Figure 1b). Given that residues 1-311 contain both a DUB domain (1-170) and a putative accessory domain/VR-1 (170-311), we tested the DUB domain alone (residues 1-177) and observed increased cleavage kinetics of K48 and K63 tetra-ubiquitin chains compared to 1-311 when the enzyme was added at 500 nM (Supplementary Figure 2a). These experiments were also carried out with $OtDUB_{1-259}$, which exhibits activity comparable to or slightly better than $OtDUB_{1-311}$ when tested against Ub-AMC, tetra-ubiquitin and di-ubiquitin at both 50 nM and 500 nM enzyme concentrations (Supplementary Figure 2a-c). Residues 260-311 were removed as they are disordered based on the APO crystal structure (Figure 1a). From these data, we hypothesized residues 170-259 contained a UBD.

To test ubiquitin-binding of this domain, we performed Superdex-75 size exclusion chromatography (SEC) with the putative $UBD_{170-264}$ and mono-ubiquitin run either separately or after co-incubation (Supplementary Figure 1c). Ubiquitin alone eluted at the expected column volume (~85 ml), while the UBD elution was not detected by UV absorption as it has no aromatic residues. When the two are complexed the ubiquitin peak shifts forward to ~75 ml and both proteins are present in the peak when analyzed by SDS-PAGE (Supplementary Figure 1c).

Supplementary Figures

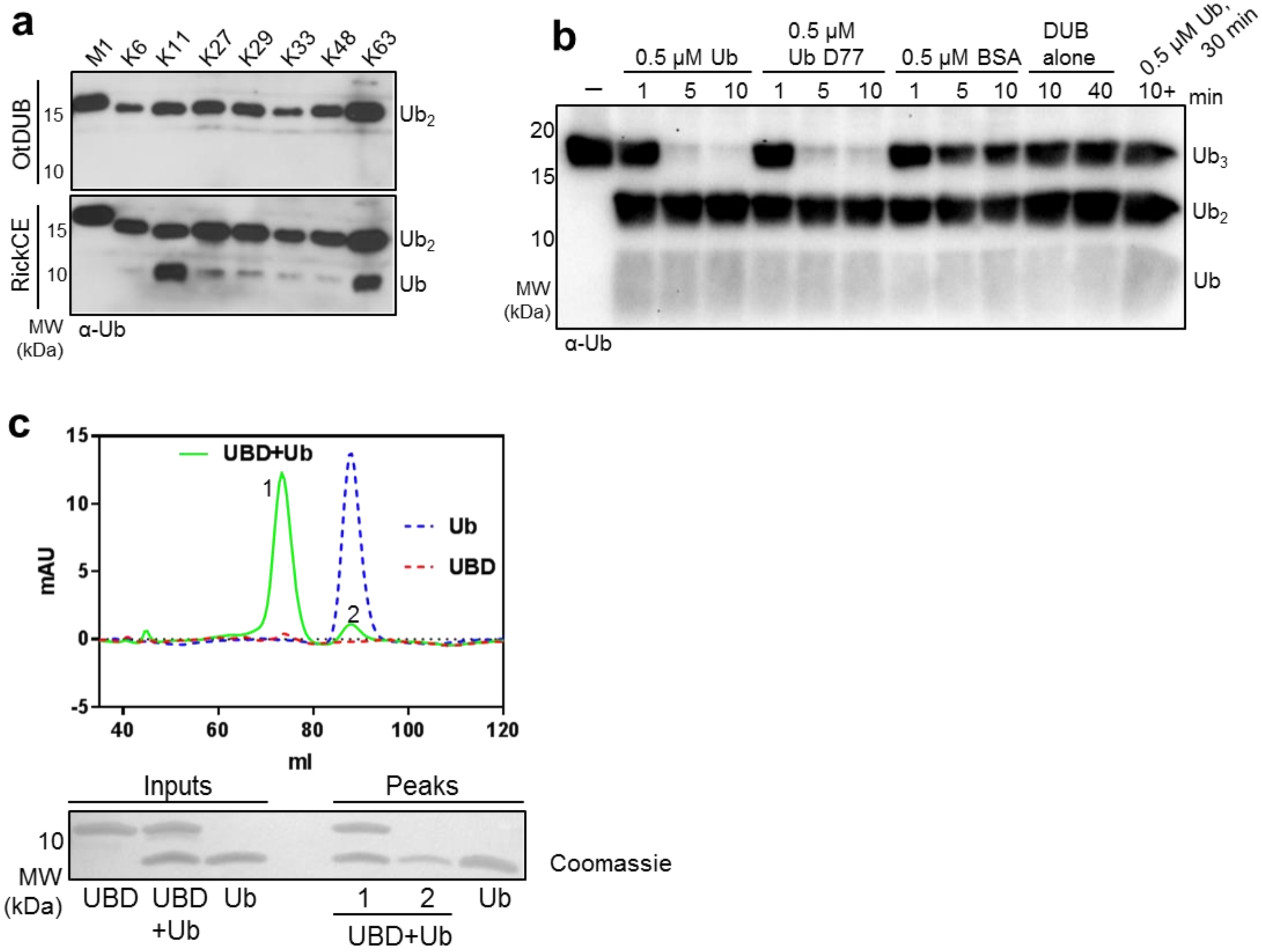

Supplementary Figure 1. $OtDUB_{1\text{-}311}$ does not detectably cleave di-ubiquitin and contains a ubiquitin binding domain. **a** Di-ubiquitin Explorer Panel (LifeSensors) was used at 1 µM to test the chain specificity of $OtDUB_{1\text{-}311}$ (0.5 µM) in a 60 min cleavage assay (*n=2*). The RickCE DUB (0.5 µM) was used as a positive control with results comparable to previously published data (*n=1*)[11]. Blots were visualized on film for maximum sensitivity and detection of cleaved ubiquitin. Source data are provided as a Source Data file. **b** Representative immunoblot of cleavage assay performed on K63 tri-ubiquitin chains with $OtDUB_{1\text{-}311}$ after preincubation with a cleavage reaction product (WT mono-Ub or Ub-D77, which carries an addition residue–Asp–after ubiquitin) versus bovine serum albumin (BSA), DUB alone or incubation with mono-ubiquitin 10 minutes after cleavage (*n=2*). All reactions were performed with 0.5 µM of the $OtDUB_{1\text{-}311}$ fragment versus 1 µM of ubiquitin chains. Source data are provided as a Source Data file. **c** Peak-shift assay showing complex formation between ubiquitin and $OtDUB_{170\text{-}264}$ (UBD) on a HiLoad Superdex 75 gel filtration column versus UBD or ubiquitin alone (4 mg/ml of each) (*n=2*). Normalized input and peak fractions were resolved on a high-percentage Tricine gel (*n=1*). Source data are provided as a Source Data file.

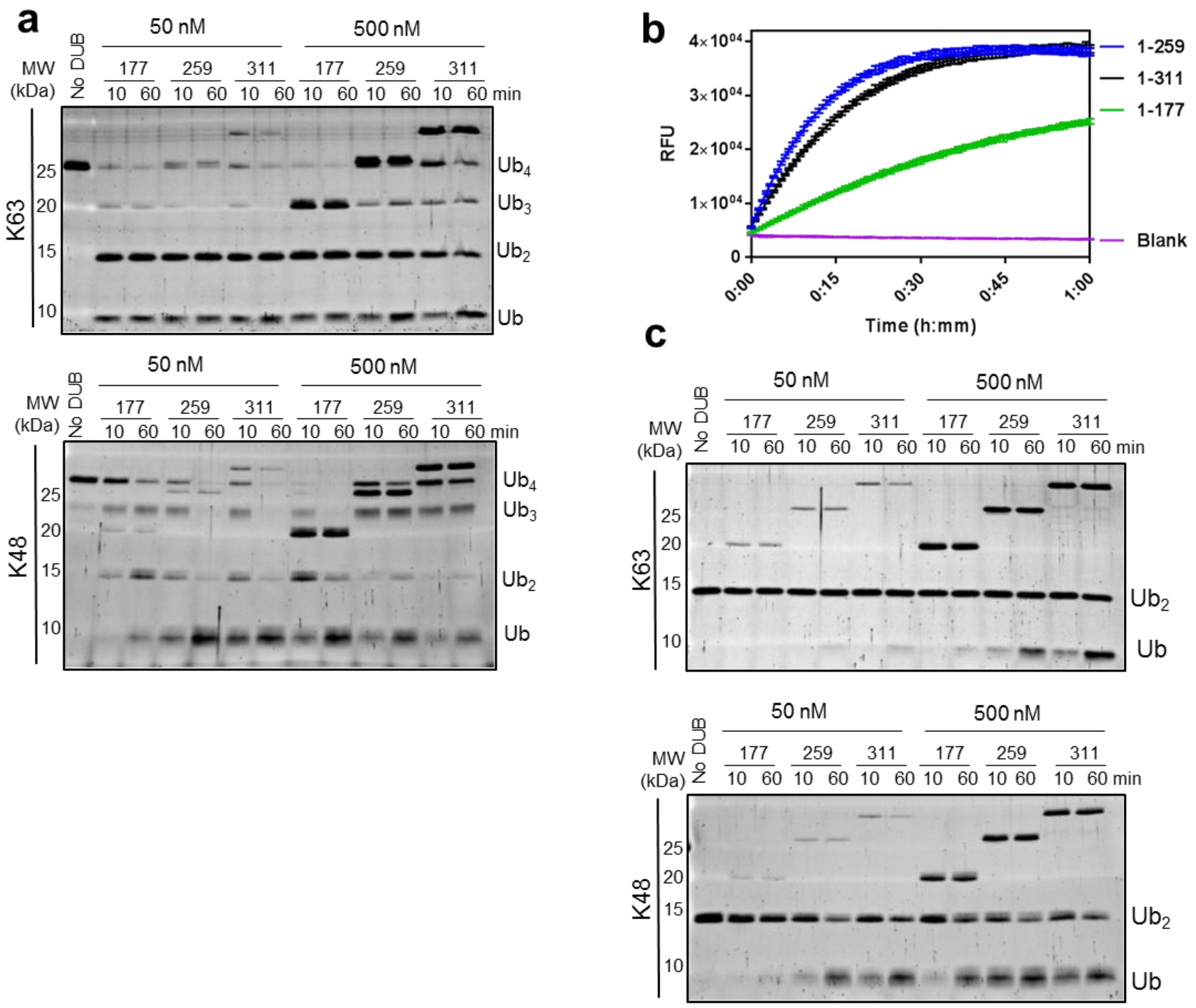

Supplementary Figure 2. OtDUB cleavage kinetics are modulated by the UBD. **a** Assessment of K63 (top) and K48 (bottom) tetra-ubiquitin cleavage by OtDUB$_{1\text{-}177}$, OtDUB$_{1\text{-}259}$ and OtDUB$_{1\text{-}311}$. Tetra-ubiquitin (1 µM) was incubated with the indicated OtDUB fragment at either 50 nM or 500 nM, resolved by SDS-PAGE and detected by SYPRO Ruby protein stain. *n*=2. Source data are provided as a Source Data file. **b** Ub-AMC cleavage assay comparing the activities of OtDUB$_{1\text{-}177}$, OtDUB$_{1\text{-}259}$ and OtDUB$_{1\text{-}311}$. Ub-AMC (400 nM) was incubated alone (Blank) or with the indicated polypeptide (350 pM) and monitored for AMC release. The lines represent the mean of technical triplicates with S.D. bars at each 40 sec time point. **c** Assessment of K63 (top) and K48 (bottom) di-ubiquitin cleavage by OtDUB$_{1\text{-}177}$, OtDUB$_{1\text{-}259}$ and OtDUB$_{1\text{-}311}$. Di-ubiquitin (1 µM) was incubated with the indicated OtDUB fragment at either 50 nM or 500 nM, resolved by SDS-PAGE and detected by SYPRO Ruby protein stain. *n*=2. Source data are provided as a Source Data file.

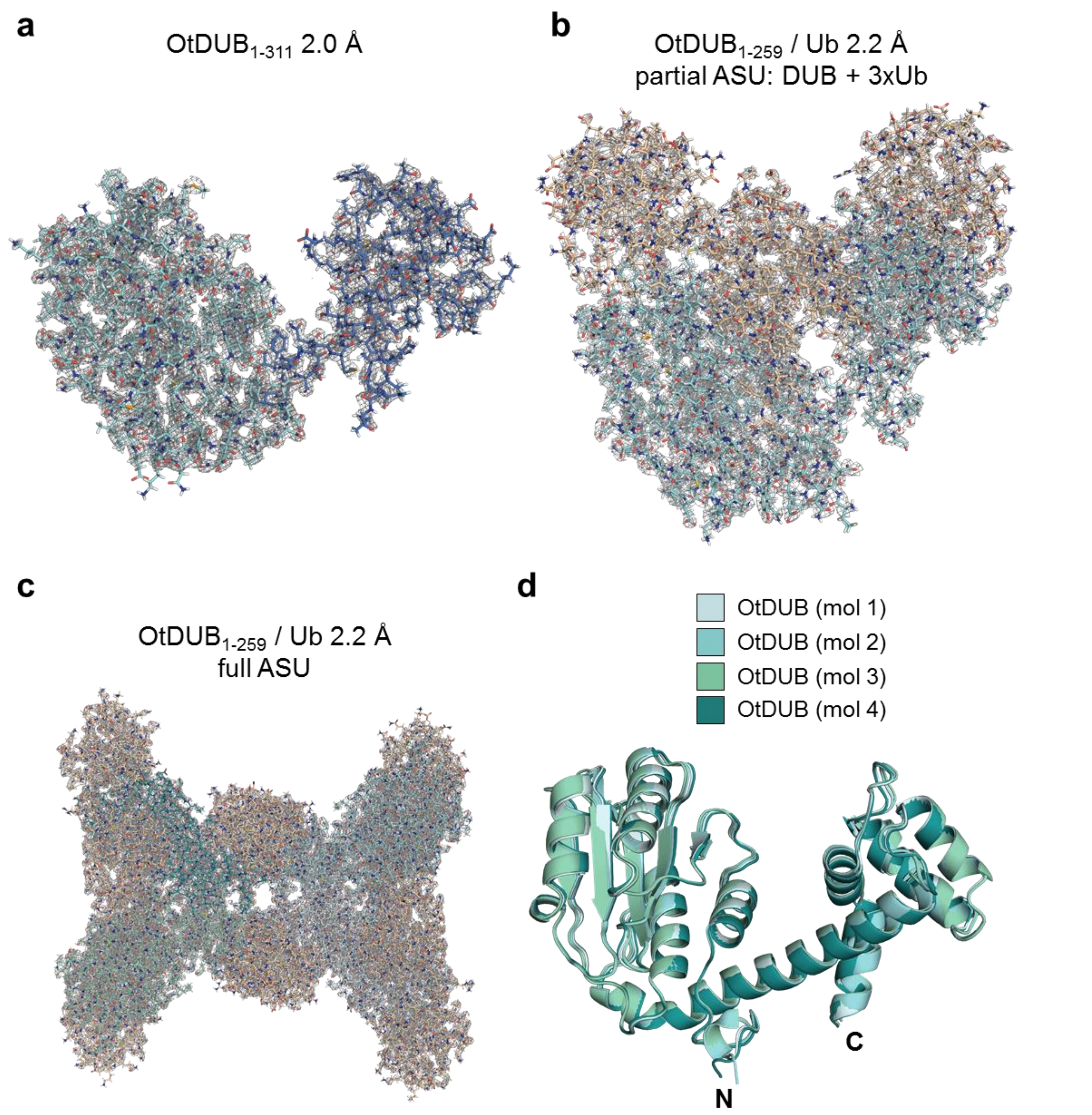

Supplementary Figure 3. Structural analysis of OtDUB apo and complexed with Ub. **a** Full asymmetric unit (ASU) of the 2.0 Å $OtDUB_{1\text{-}311}$ crystal structure showing the $2|Fo|\text{-}|Fc|$ electron density map contoured at 1σ. Catalytic DUB core is colored cyan, and VR-1/UBD is colored slate. **b** Biological assembly comprised of one molecule of $OtDUB_{1\text{-}259}$ and three molecules of Ub extracted from the 2.2 Å $OtDUB_{1\text{-}259}$ / Ub complex. The $2|Fo|\text{-}|Fc|$ electron density map is contoured at 1σ. OtDUB is colored cyan, and Ub molecules are colored tan. **c** Full ASU of the 2.2 Å $OtDUB_{1\text{-}259}$ / Ub complex crystal structure showing the $2|Fo|\text{-}|Fc|$ electron density map contoured at 1σ. OtDUB is colored in shades of cyan, and Ub is colored tan. **d** Superposition of the four copies of OtDUB in the ASU of the $OtDUB_{1\text{-}259}$ / Ub complex. The four molecules in the ASU are very similar with overall pairwise Cα RMSD between 0.2 – 0.5 Å.

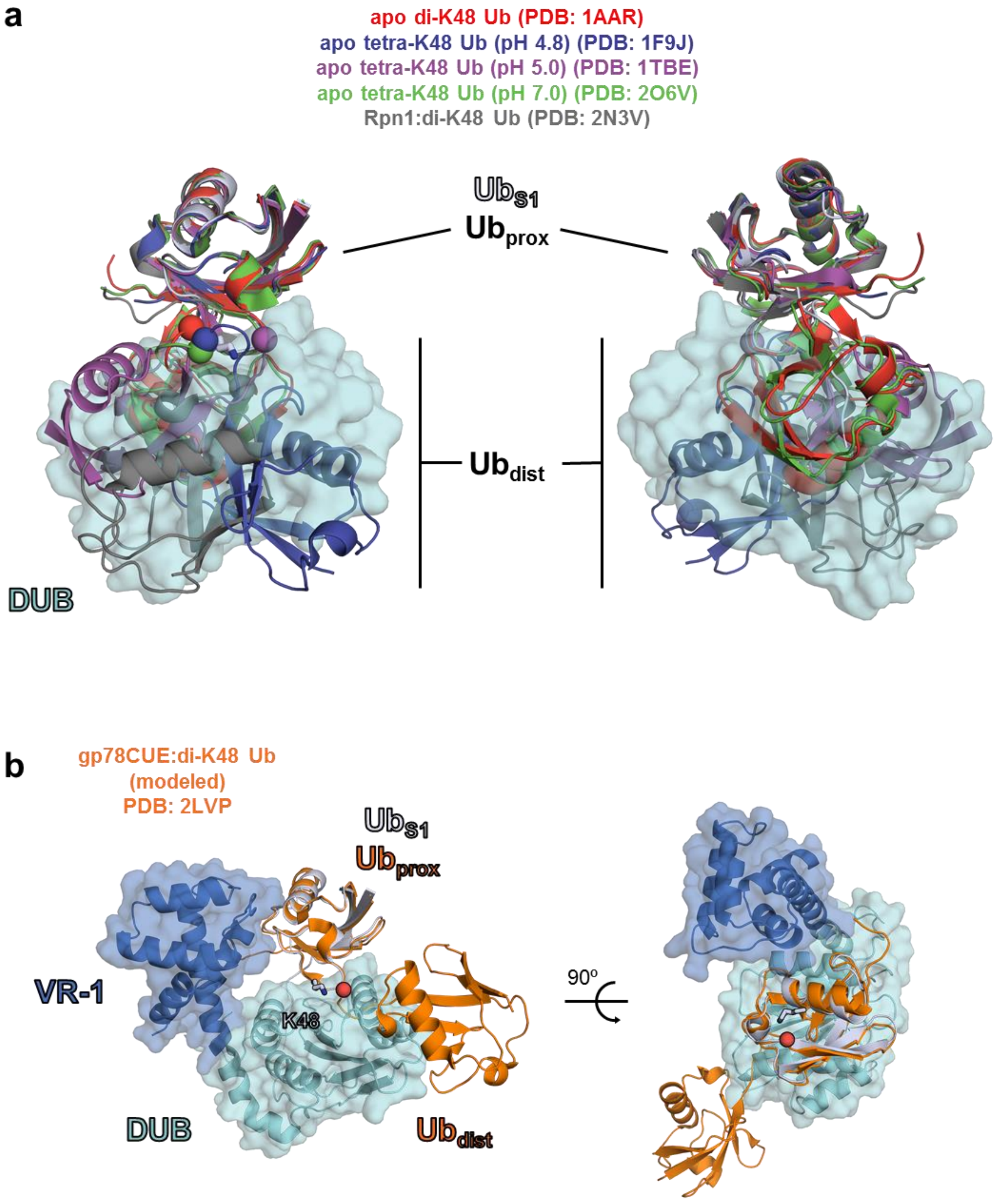

Supplementary Figure 4. Models of di-ubiquitin bound to different complexes. **a** Various K48 di-ubiquitin chains modeled into the OtDUB:ubiquitin complex. **b** K48 di-ubiquitin from the gp78:CUE domain complex modeled into the Ub$_{S1}$ site of the OtDUB:ubiquitin complex showing minimal clashing.

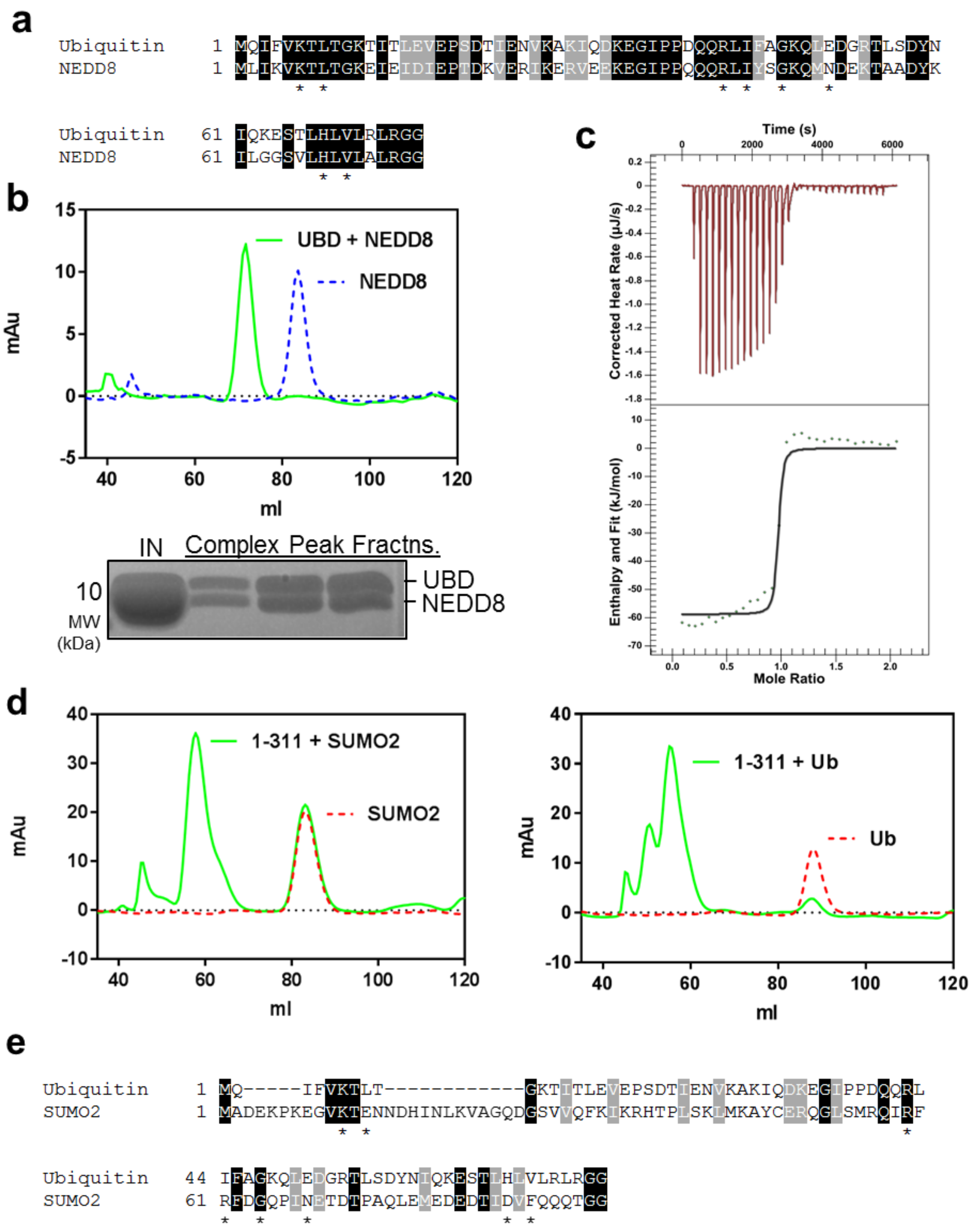

Supplementary Figure 5. The UBD of OtDUB binds to NEDD8 with high affinity and exhibits Ubl-specificity. **a** Sequence alignment of ubiquitin and NEDD8, identical residues highlighted in black, conserved residues in grey. UBD interacting residues are starred. **b** Gel filtration of NEDD8 (dashed red) alone or in complex with the OtUBD$_{170\text{-}264}$ (solid green) on a HiLoad Superdex 75 column – 200 µM of each protein (*n=2*). Input and complex peak fractions were resolved by SDS-PAGE and Coomassie stained (*n=1*). Source data are provided as a Source Data file. **c** Representative ITC titration, Upper panel shows raw injection data over time, and the lower panel shows integrated heats over the course of the reaction (*n=3*), performed with 350 µM UBD and 50 µM NEDD8. K_d = 31 ±14 nM, n = 0.9 ± 0.1. **d** Gel filtration of OtDUB$_{1\text{-}311}$ incubated together with either SUMO2 (left) (*n=1*) or ubiquitin (right) (*n=2*) at 4 mg/ml prior to loading on HiLoad Superose 75 column. **e** Sequence alignment of ubiquitin and SUMO2, identical residues highlighted in black, conserved residues in grey. UBD interacting residues are starred.

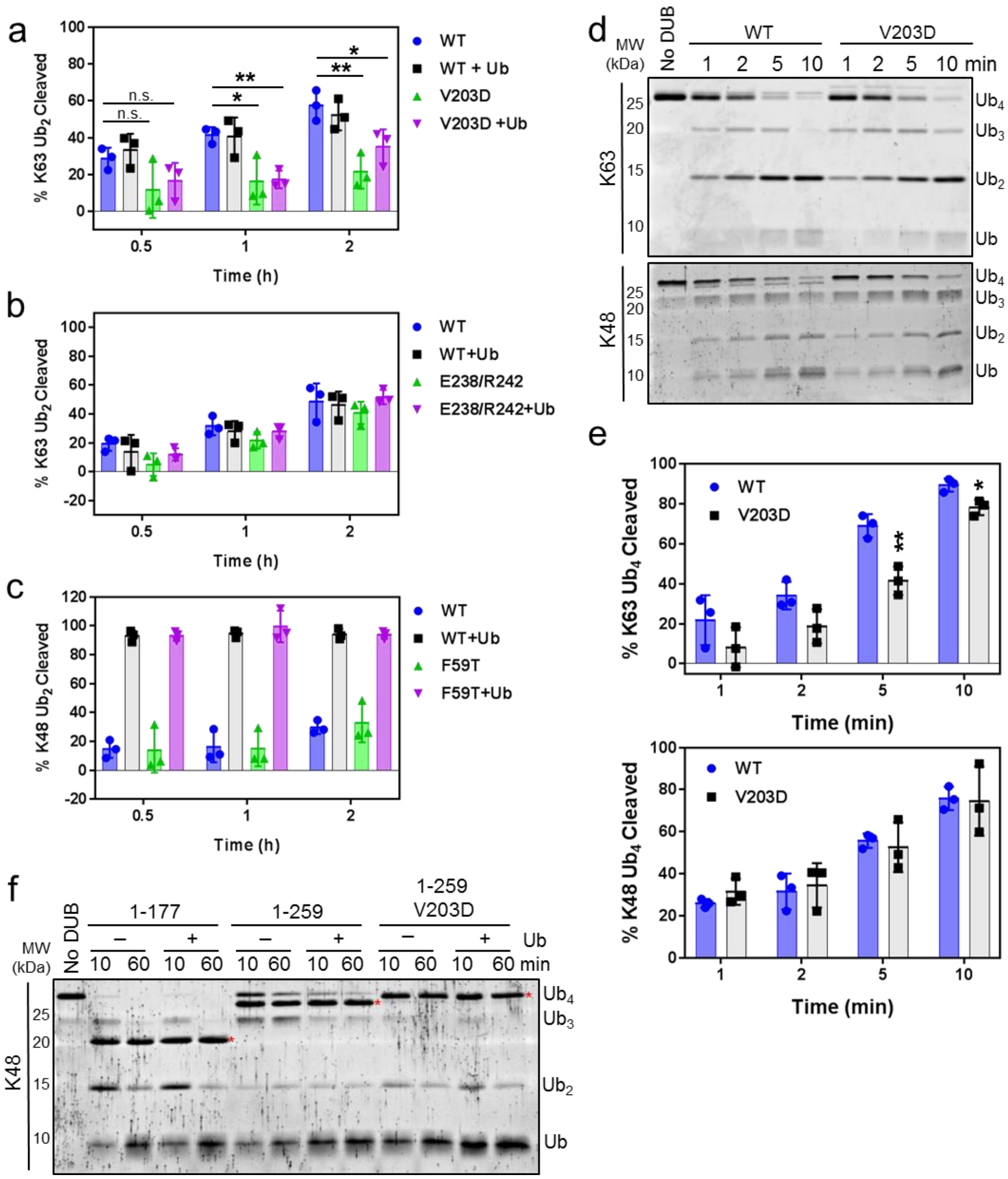

Supplementary Figure 6. The UBD modulates OtDUB activity. **a** Quantification of K63 di-ubiquitin cleavage as performed in Figure 6a. Graph shows mean and S.D. values, *n=3* independent experiments. Source data are provided as a Source Data file. **b** Quantification of K63 di-ubiquitin cleavage as performed in Figure 6c. Graph shows mean and S.D. values, *n=3* independent experiments. Source data are provided as a Source Data file. **c** Quantification of K48 di-ubiquitin cleavage as performed in Figure 6e. Graph shows mean and S.D. values, *n=3* independent experiments. Source data are provided as a Source Data file. **d** Representative tetra-ubiquitin cleavage assay using WT or V203D OtDUB$_{1-259}$. Tetra-ubiquitin K63 or K48 (2 µM) was incubated with 50 nM of the respective OtDUB for the indicated time points. *n*=3. Source data are provided as a Source Data file. **e** Quantification of K63and K48 tetra-ubiquitin (2 µM) cleavage by OtDUB$_{1-259}$ WT and V203D (50 nM). Data represent the mean of three experiments with S.D. values. Supplementary Figure 5d is a representative figure. Source data are provided as a Source Data file. **f** OtDUB fragments were preincubated with or without ubiquitin prior to initiating K48 tetra-ubiquitin cleavage. OtDUB$_{1-177}$, OtDUB$_{1-259}$, and OtDUB$_{1-259}$-V203D were preincubated with an equimolar concentration of ubiquitin before adding to 1 µM of K48 tetra-ubiquitin chains (final enzyme concentration, 0.5 µM). Samples were resolved by SDS-PAGE and detected by SYPRO Ruby protein stain. *n*=2. Red asterisks indicate enzyme protein bands. Source data are provided as a Source Data file. Unpaired, two-tailed t-tests were performed **a**, **e** for comparisons between OtDUB$_{1-259}$ WT and V203D for each condition and time point (* p<0.05, ** p <0.005, n.s. = not significant).

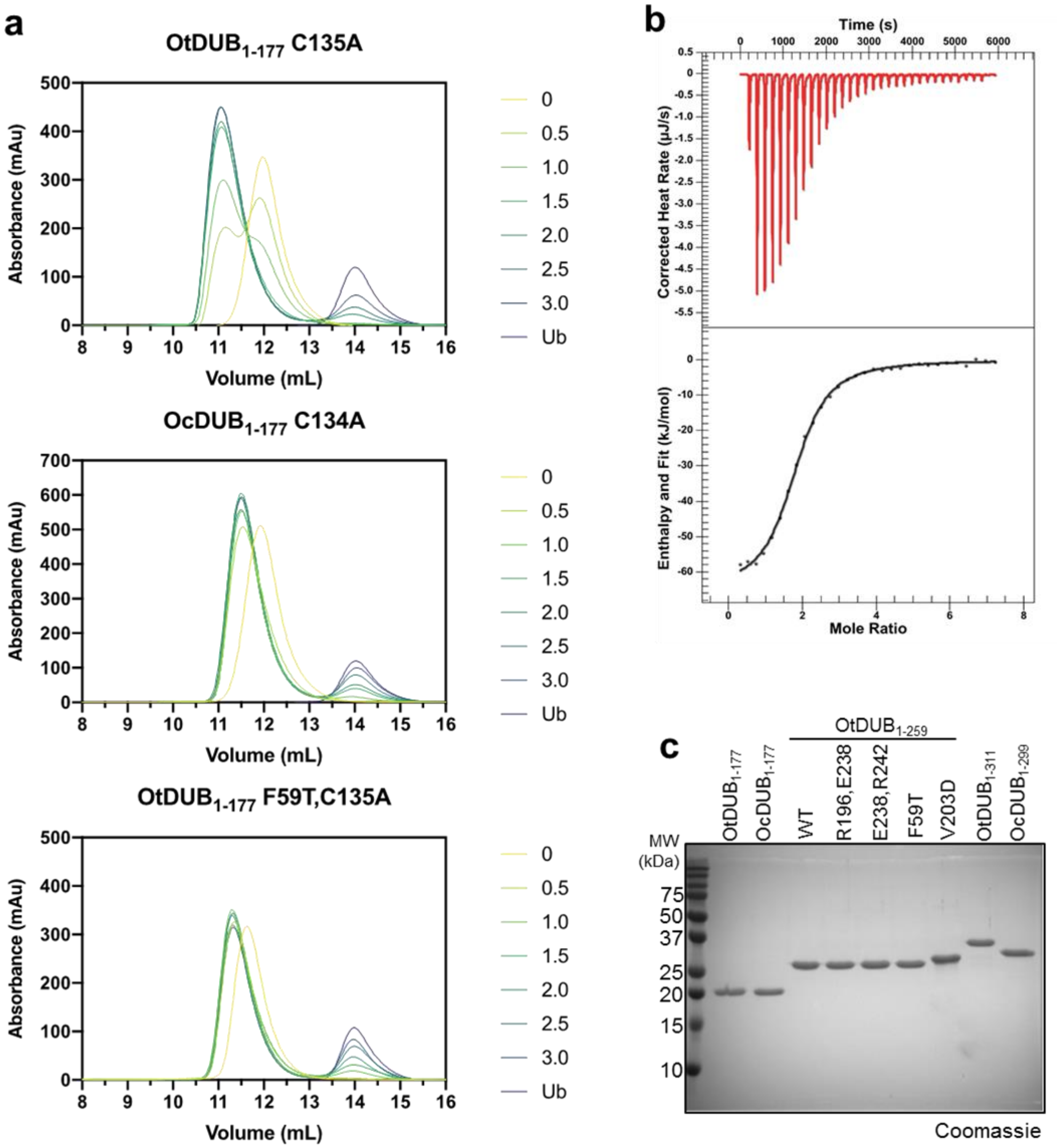

Supplementary Figure 7. OcDUB does not have an apparent S2 site and inactive OtDUB$_{1-177}$ C135A does not bind ubiquitin tightly. **a** Chromatograms for ubiquitin-titration experiments. OtDUB$_{1-177}$-C135A, OcDUB$_{1-177}$-C134A, and OtDUB$_{1-177}$-F59T,C135A were incubated with the molar equivalents of ubiquitin indicated. Complexes were resolved by SEC on a Superdex 75 column. **b** ITC of mono ubiquitin (1.25 mM) injected into 50 µM of OtDUB$_{1-177}$-C135A. *n*=3. The mean and S.D. for the derived kinetic parameters are: K_d = 6.3 µM ± 0.2, n = 1.8 ± .04, revealing weak binding of two ubiquitin molecules (S1 and S2 site occupancy). **c** Approximately 2 µg of each polypeptide used in this study for enzymatic analyses were resolved by SDS-PAGE and stained with Coomassie Blue G-250 to demonstrate their purity (*n=1*). Source data are provided as a Source Data file.
