# Supplementary material for: A deubiquitylase with an unusually high-affinity ubiquitin-binding domain from the scrub typhus pathogen Orientia tsutsugamushi
Source: Nat Commun. 2020 May 11;11:2343. doi: 10.1038/s41467-020-15985-4 (PMC7214410; doi:10.1038/s41467-020-15985-4)
Supplement: Supplementary file 3 — Description of Additional Supplementary Files [file 41467_2020_15985_MOESM3_ESM.docx]

File Name: Supplementary Data 1.

Description: Plasmids used in this study. Previously published plasmids are cited and novel plasmids include the primer sequences and cloning strategies utilized for synthesis.
