# Supplementary material for: A deubiquitylase with an unusually high-affinity ubiquitin-binding domain from the scrub typhus pathogen Orientia tsutsugamushi
Source: Nat Commun. 2020 May 11;11:2343. doi: 10.1038/s41467-020-15985-4 (PMC7214410; doi:10.1038/s41467-020-15985-4)
Supplement: Supplementary file 5 — Reporting Summary [file 41467_2020_15985_MOESM5_ESM.pdf]

nature research

Corresponding author(s): Mark Hochstrasser, Yong Xiong

Last updated by author(s): Mar 26, 2020

Reporting Summary

Nature Research wishes to improve the reproducibility of the work that we publish. This form provides structure for consistency and transparency in reporting. For further information on Nature Research policies, seeAuthors & Referees and theEditorial Policy Checklist .

Statistics

For all statistical analyses, confirm that the following items are present in the figure legend, table legend, main text, or Methods section.

n/a	Confirmed	
☐	☒	The exact sample size (n) for each experimental group/condition, given as a discrete number and unit of measurement
☐	☒	A statement on whether measurements were taken from distinct samples or whether the same sample was measured repeatedly
☐	☒	The statistical test(s) used AND whether they are one- or two-sided<br>*Only common tests should be described solely by name; describe more complex techniques in the Methods section.*
☒	☐	A description of all covariates tested
☒	☐	A description of any assumptions or corrections, such as tests of normality and adjustment for multiple comparisons
☐	☒	A full description of the statistical parameters including central tendency (e.g. means) or other basic estimates (e.g. regression coefficient) AND variation (e.g. standard deviation) or associated estimates of uncertainty (e.g. confidence intervals)
☐	☒	For null hypothesis testing, the test statistic (e.g. F, t, r) with confidence intervals, effect sizes, degrees of freedom and P value noted<br>*Give P values as exact values whenever suitable.*
☒	☐	For Bayesian analysis, information on the choice of priors and Markov chain Monte Carlo settings
☒	☐	For hierarchical and complex designs, identification of the appropriate level for tests and full reporting of outcomes
☒	☐	Estimates of effect sizes (e.g. Cohen's d, Pearson's r), indicating how they were calculated

Our web collection on statistics for biologists contains articles on many of the points above.

Software and code

Policy information about availability of computer code

Data collection	HKL2000 ver. 715 for collecting crystal diffraction data<br>FOTO/Analyst PC Image ver. 10.41 for collecting Coomassie stained gel images<br>Bio-Rad Image Lab Touch Software Version 2.2.0.08 for SYPRO Ruby stained gel data collection<br>GeneSnap 7.12 for capturing immunoblot images and Coomassie stained gel images
Data analysis	ImageJ 1.50i was used for densitrometry purposes of SYPRO Ruby stained gels. PHASER 2.8.2 used for molecular replacement, REFMAC5 5.8.0238 maximum likelihood molecular refinement, COOT 0.8.7 manual model building, Adobe Photoshop Version: 21.0.3 used for image inversion, cropping, and linear adjustments.

For manuscripts utilizing custom algorithms or software that are central to the research but not yet described in published literature, software must be made available to editors/reviewers. We strongly encourage code deposition in a community repository (e.g. GitHub). See the Nature Research guidelines for submitting code & software for further information.

Data

Policy information about availability of data

All manuscripts must include a data availability statement. This statement should provide the following information, where applicable:
- Accession codes, unique identifiers, or web links for publicly available datasets
- A list of figures that have associated raw data
- A description of any restrictions on data availability

PDB Structure IDs: 6UPU, 6UPS. Source data for all relevant figures are supplied in the Source Data File along with statistical analysis parameters. This includes: Figures 1e, 3c-d, 3f-g, 4b-c, 4e-f, 5b, 5d, 6a-e, 6g-h, 7c-e, S1a-d, S2a, S2c, S5b, S6a-f, S7c.

Field-specific reporting

Please select the one below that is the best fit for your research. If you are not sure, read the appropriate sections before making your selection.

- [x] Life sciences
- [ ] Behavioural & social sciences
- [ ] Ecological, evolutionary & environmental sciences

For a reference copy of the document with all sections, see nature.com/documents/nr-reporting-summary-flat.pdf

Life sciences study design

All studies must disclose on these points even when the disclosure is negative.

Sample size	No sample-size calculation was performed. Samples sizes were chosen as an n=3, the minimum to allow for statistical analysis and ensure reproducibility.
Data exclusions	No data were excluded from the analyses.
Replication	All attempts at replication were successful. Most experiments were repeated for at least an n=2 and in quantification cases an n=3. The exceptions are: Figure 7f was performed once. The data came out extremely clear and with the anticipated results. Further there are positive and negative controls present in the experiment. Figure S1a (RickCe data was performed once as a control to OtDUB data performed twice and was comparable to previously published data). Figure S1c, S4b bottom panels (the peak shifts were performed twice [top panels]). Since the peak shift assays were identical it seemed unnecessary to rerun fractions for visualization. Figure S4d. The peak shift assay with OtDUB 1-311 and human SUMO2 was performed once and showed negative data compared to binding ubiquitin, which was repeated twice with the same batch of OtDUB 1-311 protein. This result is also consistent with the lack of conserved UBD binding residues in SUMO2 (Figure S4e). Figure S6c is a simple control experiment performed once to exhibit protein purity, therefore was not necessary to repeat.
Randomization	This is not relevant to this study as it is unnecessary for the experiments performed. There are no treatment groups to randomly choose from for the quantified cleavage assays.
Blinding	It is not possible to do such biochemical experiments blindly without jeopardizing the integrity of the reagent allocation to each tube or reaction. This is never done.

Reporting for specific materials, systems and methods

We require information from authors about some types of materials, experimental systems and methods used in many studies. Here, indicate whether each material, system or method listed is relevant to your study. If you are not sure if a list item applies to your research, read the appropriate section before selecting a response.

Materials & experimental systems

n/a	Involved in the study	
☐	☒	Antibodies
☒	☐	Eukaryotic cell lines
☒	☐	Palaeontology
☒	☐	Animals and other organisms
☒	☐	Human research participants
☒	☐	Clinical data

Methods

n/a	Involved in the study	
☒	☐	ChIP-seq
☒	☐	Flow cytometry
☒	☐	MRI-based neuroimaging

Antibodies

Antibodies used	Rabbit polyclonal antibody against ubiquitin, Dako Ref # Z0458, Lot # 20016967<br>Anti-Rabbit IgG, HRP linked antibody from donkey GE Healthcare Ref # NA934V, Lot # 16918036
Validation	Cited in at least 89 publications for various applications, including western blotting as used in this manuscript https://www.labome.com/product/Dako/Z0458.html
